# Monitoring scanner calibration using the image-derived arterial blood SUV in whole-body FDG-PET

**DOI:** 10.1186/s13550-018-0391-7

**Published:** 2018-05-15

**Authors:** Jens Maus, Frank Hofheinz, Ivayla Apostolova, Michael C. Kreissl, Jörg Kotzerke, Jörg van den Hoff

**Affiliations:** 1 Helmholtz-Zentrum Dresden-Rossendorf, PET Center, Institute of Radiopharmaceutical Cancer Research, Bautzner Landstraße 400, Dresden, Germany; 2 Zentrum für Radiologie und Endoskopie, Abteilung für Nuklearmedizin, Universitätsklinikum Hamburg-Eppendorf, Hamburg, Germany; 3 Klinik für Radiologie und Nuklearmedizin, Universitätsklinikum Magdeburg A.ö.R., Magdeburg, Germany; 4 Klinik und Poliklinik für Nuklearmedizin, Universitätsklinikum Carl Gustav Carus, Dresden, Germany

**Keywords:** PET, Quantification, Blood SUV, Standardization, Multicenter, In vivo

## Abstract

**Background:**

The current de facto standard for quantification of tumor metabolism in oncological whole-body PET is the standardized uptake value (SUV) approach. SUV determination requires accurate scanner calibration. Residual inaccuracies of the calibration lead to biased SUV values. Especially, this can adversely affect multicenter trials where it is difficult to ensure reliable cross-calibration across participating sites. The goal of the present work was the evaluation of a new method for monitoring scanner calibration utilizing the image-derived arterial blood SUV (BSUV) averaged over a sufficiently large number of whole-body FDG-PET investigations.

Data of 681 patients from three sites which underwent routine ^18^F-FDG PET/CT or PET/MR were retrospectively analyzed. BSUV was determined in the descending aorta using a three-dimensional ROI concentric to the aorta’s centerline. The ROI was delineated in the CT or MRI images and transferred to the PET images. A minimum ROI volume of 5 mL and a concentric safety margin to the aortic wall was observed. Mean BSUV, standard deviation (SD), and standard error of the mean (SE) were computed for three groups of patients at each site, investigated 2 years apart, respectively, with group sizes between 53 and 100 patients. Differences of mean BSUV between the individual groups and sites were determined.

**Results:**

SD (SE) of BSUV in the different groups ranged from 14.3 to 20.7% (1.7 to 2.8%). Differences of mean BSUV between intra-site groups were small (1.1–6.3%). Only one out of nine of these differences reached statistical significance. Inter-site differences were distinctly larger (12.6–25.1%) and highly significant (*P*<0.001).

**Conclusions:**

Image-based determination of the group-averaged blood SUV in modestly large groups of whole-body FDG-PET investigations is a viable approach for ensuring consistent scanner calibration over time and across different sites. We propose this approach as a quality control and cross-calibration tool augmenting established phantom-based procedures.

## Background

The current de facto standard for quantitative assessment of tumor metabolism in oncological whole-body ^18^F-2-fluoro-2-deoxy-D-glucose (FDG) positron emission tomography (PET) is the use of the standardized uptake value (SUV) [1–3] which equals the tracer concentration in the targeted tumor/tissue normalized to the injected dose per unit body weight. SUV computation thus requires quantitative determination of the tracer concentration with PET which in turn necessitates a regularly performed thorough scanner calibration (and cross-calibration against a dose calibrator) based on suitable phantom measurements adhering to standardized procedures [2, 4–6]. Residual inaccuracies of the calibration procedure or inadequacies that manifest themselves only in vivo lead to SUV values which might be systematically biased relative to the true values. Especially, this can adversely affect multicenter trials [7]. In such trials, additional phantom measurements are commonly performed to check whether calibration differences across the different involved scanners remain within tight limits so that SUVs derived at different sites are consistent and can be pooled during evaluation of the trial data [8, 9]. Otherwise, existing systematic calibration differences between sites need to be corrected by suitable cross-calibration factors between the different centers/PET systems to enforce data consistency [10].

One practical problem in this context is that such repeated phantom measurements cause additional workload during execution of the study. Moreover, in between consecutive calibration measurements, there is no guarantee that the respective PET system does not exhibit a certain drift in efficiency for assorted reasons, potentially invalidating the given calibration [7].

It would, therefore, be desirable to have an easy method for monitoring the calibration of a PET system in order to exclude temporal drifts prior to the next calibration, providing also a consistency check across successive calibrations. In a multicenter setting, such a method could also allow determining the effective cross-calibration factors between different PET systems. This could be especially useful because these factors might deviate significantly from the ideally expected value (i.e., one) despite all efforts at ensuring accurate absolute calibration of all involved PET systems.

One possible solution would be to use the recently proposed approach [11] to compare activity concentrations of urine samples measured in a well counter with activity concentrations extracted from PET images of the bladder region. In this study, we could show that this method is able to identify differences in in vivo quantification accuracy (the ability of PET to reproduce the well counter results) across different PET scanners at the 5% accuracy level [12].

While accuracy of this method would be adequate for monitoring scanner calibration, it obviously creates additional work in terms of data collection (urine sampling, well counter measurement, possibly additional PET scan across bladder) and handling which makes it unattractive for clinical routine and also for multicenter trials where the quality assurance tasks are already quite challenging [9, 13].

In the present paper, we, therefore, propose a more practicable alternative. We start from the observation that the SUV approach to quantify tumor metabolism ultimately rests on the assumption that the arterial input function when measured in SUV units is essentially invariant across different patients. Although this assumption is not fulfilled exactly [14, 15], it is still true that the blood SUV (BSUV) at a given time after FDG injection exhibits only moderate inter-subject variability following an approximately Gaussian distribution around the population mean [16, 17]. Since this variability is rather small, it should be possible to quite accurately estimate this population average from modest group sizes and to interpret statistically significant differences in group averages as being due to differences in scanner calibration rather than as differences of true mean BSUV between different groups. In this paper, we investigate the viability of this approach.

## Methods

### Patient group and data acquisition

For a total of 681 whole-body FDG-PET scans from three different institutions, BSUV was determined using the procedure explained in detail further below. Two hundred fifty-three measurements were performed on a Siemens Biograph 16 PET/CT, 252 measurements on a Philips Ingenuity-TF PET/MR, and 176 measurements on a Siemens Biograph mCT PET/CT scanner. For further details on the used PET systems and image reconstruction, see Table 1.

**Table 1:** Used PET systems and image reconstruction details

	Site 1	Site 2	Site 3
Scanner type	Biograph 16 PET/CT, Siemens Medical Solutions, Knoxville, TN, USA	Ingenuity-TF PET/MR, Philips Healthcare, Best, The Netherlands	Biograph mCT 64 PET/CT, Siemens Medical Solutions, Knoxville, TN, USA
PET calibration procedure	Vendor specific	Vendor specific	EARL certified
Residual error	< 3%	< 4%	< 5%
Scan duration per bed position [min]	3	2	3
Reconstruction method	OSEM	BLOB-OS TF	PSF+TOF
Iterations/subsets	4i/8s	3i/32s	2i/21s
PET			
Matrix size	168×168	144×144	200×200
Voxel size	4.1×4.1×5.0 mm^3^	4.0×4.0×4.0 mm^3^	4.1×4.1×3.0 mm^3^
Filter (FWHM)	Gaussian (5 mm)	n/a	Gaussian (5 mm)
Attenuation correction	CTAC	MRAC (non-TC)	CTAC
CT / MR	CT	MR	CT
Matrix size	512×512	320×320	512×512
Voxel size	1.4×1.4×5.0 mm^3^	1.9×1.9×6.0 mm^3^	1.5×1.5×3.0 mm^3^

The scans were performed between 2011 and 2017. Included were ^18^F-FDG whole-body PET/CT or PET/MR scans independent of clinical indication. The only exclusion criterion was the presence of strong artifacts in the attenuation CT or MRI.

The pooled data from each site were used for inter-site comparisons. To allow intra-site comparisons, for each site, the data were divided into three groups of consecutive scans from time windows approximately 2 years apart; see Table 2 and Fig. 3. The required minimal group size was *N*=50.

**Table 2:** Description of patient groups (values expressed as mean ± standard deviation)

	All	Group 1	Group 2	Group 3
Site 1				
Number of patients	253 (186m, 67f)	100 (70m, 30f)	53 (50m, 3f)	100 (66m, 34f)
Age [years]	61.9 ± 13.9	61 ± 13.4	58.4 ± 12.9	64.6 ± 14.5
Weight [kg]	75.9 ± 15.7	77.2 ± 16.5	76 ± 13.7	74.7 ±16.1
Dosage [MBq]	349 ± 40	326 ± 26	332 ± 45	380 ±27
Scan start p.i. [min]	74 ± 15	71 ± 12	77 ± 15	76 ± 17
Site 2				
Number of patients	252 (154m, 98f)	98 (60m, 38f)	100 (62m, 38f)	54 (32m, 22f)
Age [years]	55.1 ± 16.7	57.7 ± 15.3	51.2 ± 17.2	57.9 ± 17.3
Weight [kg]	76.8 ± 16.5	79.3 ± 14.8	74.3 ± 16.8	76.6 ± 18.3
Dosage [MBq]	298 ± 25	297 ± 22	299 ± 29	299 ± 19
Scan start p.i. [min]	76 ± 18	74 ± 15	77 ± 20	76 ± 17
Site 3				
Number of patients	176 (131m, 45f)	55 (45m, 10f)	59 (41m, 18f)	62 (45m, 17f)
Age [years]	64.8 ± 11.6	66.3 ± 9.2	63.8 ± 12.9	64.3 ± 12.2
Weight [kg]	79.2 ± 15.6	78.6 ± 14.9	79.2 ± 16.6	79.8 ± 15.5
Dosage [MBq]	234 ± 11	235 ± 9	232 ± 8	234 ± 14
Scan start p.i. [min]	64 ± 8	66 ± 6	66 ± 11	59 ± 4

### Data analysis/ROI definition

**Fig. 1:**
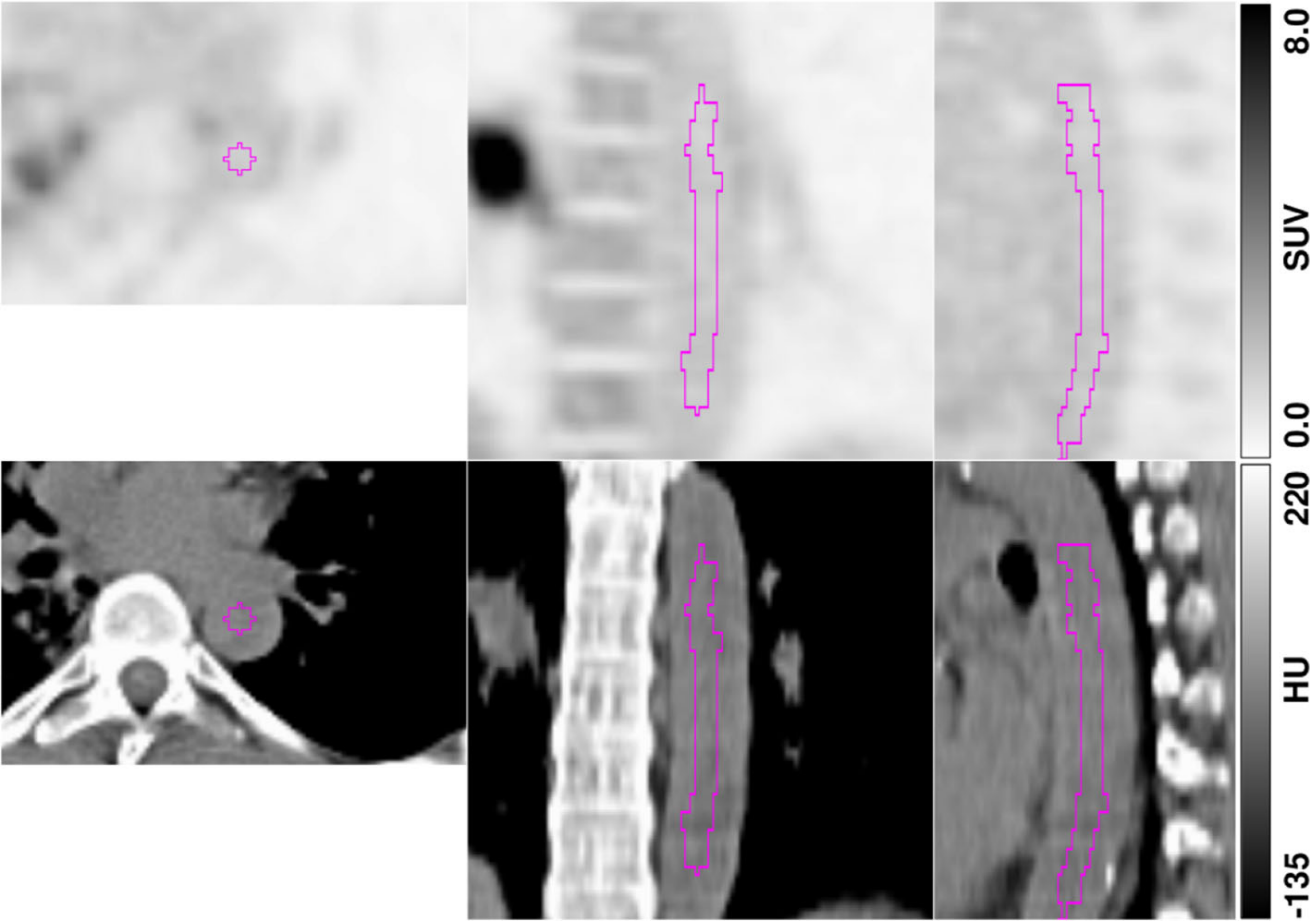
Example of aorta ROI delineation (top, PET; bottom, CT). Delineation was performed in the transaxial CT slices and transferred to the PET image volume. Shown are orthogonal slices through the 3D-ROI (magenta line)

For determination of the arterial blood SUV, a three-dimensional region of interest (ROI), concentric to the aorta’s centerline, was delineated in the attenuation CT or MRI and copied to the corresponding PET data. The ROI was positioned in the descending thoracic aorta. Planes showing either high tracer uptake in the immediate vicinity of the aorta or exhibiting obvious attenuation/scatter correction artifacts were excluded. A minimum ROI volume of 5 mL was chosen to ensure sufficient statistical accuracy. In order to avoid partial volume effects, a concentric safety margin of ≈ 8 mm distance to the aortic wall was maintained. One example of such an aorta delineation is shown in Fig. 1. BSUV(*T*) was computed as the average SUV in the aorta ROI (*T* uptake time p.i.).

The described procedure essentially avoids partial volume effects (spill out as well as spill in) even in case of an obvious eccentric positioning of the ROI within the aorta. This, in turn, ensures low inter-observer (and even smaller intra-observer) variability of the resulting ROI average value. In our experience, inter-observer variability is characterized by an absence of systematic bias and a standard deviation of less than 4%.

Intra-scan differences in uptake time were accounted for by referring all values to a common reference time according to

1 $$  \text{BSUV} = \text{BSUV}(T_{0}) = \text{BSUV}(T) \times \left(\frac{T}{T_{0}}\right)^{b}\,,  $$

where *T*_0_=75 min is the chosen reference time and *b*=0.313. This equation follows from a previous study [15] according to which the time dependence of BSUV in the relevant time window can be described by the inverse power law:

2 $$ \text{BSUV}(T) \propto\,\frac{1}{T^{b}}\,.  $$

### Data evaluation

Inter- and intra-site group differences were tested for significance using a two-sided *t* test.

For each group, the mean BSUV, standard deviation (SD), and standard error of the mean $\text {SE} = \text {SD}/\sqrt {N}$ (*N*: group size) were computed. Group differences of mean BSUV were computed as

3 $$ \Delta\text{BSUV} = 2 \times \frac{|\text{BSUV}_{A} - \text{BSUV}_{B}|} {\text{BSUV}_{A} + \text{BSUV}_{B}}\,.  $$

Statistical analysis was performed using the *R language and environment for statistical computing* version 3.4.3 [18]. ROI delineations were performed using the software *ROVER*, version 3.0.22 (ABX GmbH, Radeberg, Germany).

## Results

**Fig. 2:**
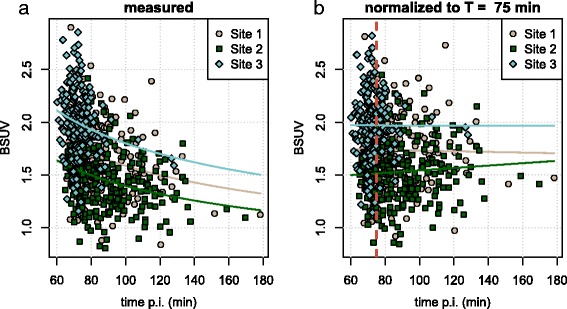
BSUV as a function of uptake time. **a** Measured values. Solid lines represent least squares fits according to Eq. 1 (when solving that equation for BSUV(*T*) and fitting for BSUV(*T*_0_)). **b** Values normalized to reference time *T*_0_=75 min (dashed red line). Solid lines represent least squares fits of straight lines to these data

BSUV values (measured and normalized to *T*=75 min) are shown in Fig. 2. As expected, there is a slight decrease of measured BSUV over time (*P*<0.02) which is essentially removed after normalization.

**Fig. 3:**
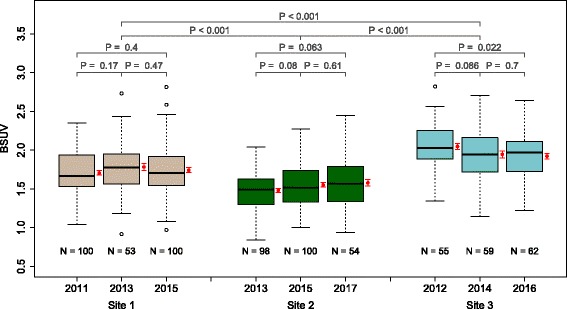
Boxplot of the derived BSUV distributions in the different patient groups and sites. Significance levels for group and site differences are indicated. Group averaged BSUV is shown in red beside the respective box (error bars represent the standard error of the mean)

The main results are summarized in Table 3 and Fig. 3. In view of the relatively small inter-subject variability (SD between 14.3 and 20.7%), the mean BSUV could be determined with high statistical accuracy in all patient groups for the chosen group sizes (SE between 1.7 and 2.8%). Intra-site group differences of mean BSUV did not exceed 6.3% and were statistically insignificant with one exception (site 3, group 1 vs. 3); see Table 4. Mean BSUV differences between the three sites were notable (12.6 to 25.1%) and highly significant (*P*<0.001); see Table 5.

**Table 3:** Group-averaged BSUV and corresponding absolute and relative standard deviations (SD) and standard errors of the mean (SE) for the different patient groups and sites N: group size

	N	BSUV	SD	SE	SD (%)	SE (%)
Site 1						
Group 1	100	1.71	0.28	0.028	16.6	1.7
Group 2	53	1.78	0.35	0.048	19.5	2.7
Group 3	100	1.74	0.31	0.031	17.9	1.8
All	253	1.74	0.31	0.019	17.8	1.1
Site 2						
Group 1	98	1.48	0.26	0.027	17.8	1.8
Group 2	100	1.55	0.29	0.029	18.9	1.9
Group 3	54	1.58	0.33	0.044	20.7	2.8
All	252	1.53	0.29	0.018	19.0	1.2
Site 3						
Group 1	55	2.05	0.29	0.040	14.3	1.9
Group 2	59	1.95	0.34	0.045	17.6	2.3
Group 3	62	1.92	0.29	0.037	15.2	1.9
All	176	1.97	0.31	0.024	15.9	1.2

**Table 4:** Intra-site differences of group-averaged BSUVs

	*Δ*BSUV (%)	*P* value
Site 1		
Group 1 vs. group 2	4.40	*P*=0.17
Group 2 vs. group 3	2.33	*P*=0.47
Group 1 vs. group 3	2.07	*P*=0.40
Site 2		
Group 1 vs. group 2	4.59	*P*=0.08
Group 2 vs. group 3	1.76	*P*=0.61
Group 1 vs. group 3	6.35	*P*=0.063
Site 3		
Group 1 vs. group 2	5.17	*P*=0.086
Group 2 vs. group 3	1.15	*P*=0.70
Group 1 vs. group 3	6.33	*P*=0.022

**Table 5:** Differences of site-averaged BSUVs

	*Δ*BSUV (%)	*P* value
Site 1 vs. site 2	12.61	*P*<0.001
Site 2 vs. site 3	25.09	*P*<0.001
Site 1 vs. site 3	12.58	*P*<0.001

## Discussion

The main result of this investigation is that determination of the group-averaged image-derived BSUV in whole-body FDG-PET is possible with quite high statistical accuracy of 1.7–2.8% in moderately large (*N*≈50−100) patient groups. This is a direct consequence of the fact that inter-subject variability of BSUV is modest (≲ 20% SD) [16, 17].

For each of the three sites included in the present study, the selection of three patient groups scanned in time windows separated approximately by 2 years, respectively, allowed to demonstrate that mean BSUV is reproducible within rather tight limits (distinctly better than 10%). This supports the notion that the calibration procedures implemented at these sites were adequate to maintain reproducible quantification across repeated calibration and over substantial times. In fact, only a single difference (6.3% difference between group 1 vs. group 3 at site 3) reached statistical significance (*P*=0.022). This might thus represent a real change of the calibration which, however, is still well below the frequently recommended < 10 % best-practice boundary [2, 4, 5].

Since clinical PET sites generally perform several whole-body FDG-PET investigations per day, it would be conceivable to maintain a rather tight control regarding stable scanner calibration by continuously monitoring the stability of BSUV over time (50 new FDG scans should usually be acquired within 2–3 weeks). If the PET system has been absolutely calibrated once in the usual way (via phantom measurements), tracking of the mean BSUV over time would even allow, in principle, to cut down on the number of recalibrations as there would be no need for recalibration if measured BSUV does not change notably/significantly over time. The method also seems perfectly suited to detect inconsistencies between successive calibrations since BSUV should not change notably after a recalibration is performed.

Due to the achievable high statistical accuracy of the group averages, there is another interesting application for utilization of BSUV measurements, namely, enforcing consistent quantification across different (and differently calibrated) PET systems by determining the respective cross-calibration factors. In the present study, this is demonstrated by detection of notable inter-site differences in the range of 12.6–25.1%. Uncorrected differences of this magnitude are unacceptable for multicenter trials [10, 19].

Regarding the underlying cause, it is probable that the observed inter-site differerences do not solely reflect hidden errors of the phantom-based calibration measurements at any of the sites but also demonstrate (scanner dependent) limitations of validity of the phantom-based calibration for in vivo investigations (influence of attenuation and scatter correction as well as image reconstruction details) on top of which other sources of error (clock synchronization, dose calibrator, adherence to SOPs, systematically wrong determination of patient weight, etc.) might play a role. The proposed procedure of comparing group-averaged BSUV thus offers a means for assessing the “effective” SUV calibration as it is operational under in vivo conditions in patient investigations.

It goes without saying that for prospective studies, it should be demanded that all participating sites should strive to achieve accurate absolute calibration of their respective PET system. But even then, there, of course, will be some systematic quantification bias between sites. Determining this bias via additional dedicated phantom measurements is possible in principle, but time-consuming and tedious [7, 9].

We propose that group-averaged BSUV assessment is very well suited to determine the relevant cross-calibration factors which can enforce quantification consistency across different PET sites. Especially attractive is the fact that this procedure can also be applied when there is interest in retrospective quantitative evaluation of pooled data from different sites, e.g., survival analysis using a certain SUV threshold for group separation.

The detected inter-site differences of mean BSUV can be partly compared to results of our previous studies [11, 12] which included patient data from different patients but the same time period. There, we compared activity concentration in the bladder with the concentration in urine samples from two of the sites contributing also to the present investigation. In fact, both approaches yield consistent results: the inter-site differences between group 1, site 1 (Biograph 16 PET/CT) and group 1, and site 2 (Ingenuity-TF PET/MR) of 14.1% in the present study is in perfect agreement with the 12% difference seen previously [12].

The usefulness of BSUV monitoring as a method of quality control and cross-calibration between different PET systems, of course, depends on the ability to perform BSUV measurements easily and accurately. In our experience, using software suitable for 3D ROI delineation, it takes only a few minutes to process an image data set. Furthermore, it is easy to achieve high inter- and intra-observer reproducibility if the required precautions regarding minimum ROI volume, safety margin, etc.—as outlined in the “Methods” section—are observed. It might also be emphasized that, when adhering to the described procedure, the BSUV determination is not only highly reproducible but also accurate in the sense that it is not affected by partial volume (PV) effects in any relevant way. Even in a worst-case scenario (combining a very small aorta of 20 mm diameter with a very low spatial resolution of 8 mm FWHM), the ROI mean would exhibit just a 1.8% underestimate of true activity for a ROI of 4 mm diameter concenctric to the aorta’s center line. For more typical (somewhat larger) aorta diameters and somewhat better spatial resolution, PV effects are totally negligible. Especially, it is thus not relevant if the compared scanners exhibit different spatial resolution due to hardware or image reconstruction differences.

As already stated, the suitability of tumor SUV as a reliable surrogate for the tumor’s metabolic rate of glucose consumption principally rests on the implied assumption that the time-dependent tracer supply expressed in SUV units, i.e., the arterial input function BSUV (*T*) (and thus, especially, BSUV at any fixed time p.i.), can be considered to be identical across different investigations. However, the data presented in this investigation confirm previous findings that, in fact, inter-individual BSUV variability amounts to ≈± 20% (SD). This is far from being negligible and poses a problem for the SUV approach in FDG-PET.

While the presented approach to monitor group-averaged BSUV to account for a potential drift in scanner calibration or inter-site calibration differences can eliminate systematic bias from tumor SUV evaluations, it obviously is not able to account for actual inter-individual differences of BSUV (causing spurious differences in lesion uptake). In this context, it seems worthwhile to note that the recently proposed and evaluated tumor-to-blood standard uptake ratio (SUR) approach [14, 15, 17] accounts for such true inter-individual BSUV differences. There is increasing evidence that SUR is superior to SUV as a surrogate for tumor metabolism in whole-body FDG-PET [20–22]. Naturally, given its definition as an image-derived ratio, changes/differences in scanner calibration are implicitly accounted for as well. When adopting the SUR approach, the procedure evaluated in the present study would, therefore, offer no additional benefit for clinical FDG-PET. Nevertheless, it would still offer an easy way to verify stable scanner calibration and enable consistent cross-calibration across sites for studies applying the SUV approach to tracers beyond FDG.

This retrospective study has the principal limitation that no blood samples were acquired. There is thus no unambiguous proof that the observed inter-site differences of BSUV group averages are reflecting differences in effective scanner calibration (including all possible effects of imperfect attenuation correction and image reconstruction of the patient scans). One could hypothesize that the observed BSUV group differences are in fact partly or completely reflecting physiological differences between the respective patient groups. While this possibility cannot be ruled out completely, we think this to be highly unlikely for the following reasons.

As with any other physiological parameters (e.g., blood glucose level, blood pressure), BSUV follows a certain unknown distribution function in the total population. For any representative sample drawn from the population, the sample mean and variance are unbiased best estimates of the population mean and variance (for a normal distribution, anyway). The question thus reduces to whether the different patient groups can be considered as representative samples of the total population of “all patients receiving clinical FDG-PET at different times and sites.” Given the fact that the included patients were randomly selected independent of clinical indication, we believe that this assumption can be considered fulfilled. Regarding patient preparation, too, there is not much room for adverse effects of group-specific differences, e.g., in the present study, patients at site 3 routinely received a diuretic prior to scanning while this was not the case at the other two participating sites. One could suspect that the increased urine excretion in the diuretic-receiving group might be accompanied by a notable (rather than marginal) increase in the fraction of FDG excreted prior to scanning. This, in turn, would result in a systematic decrease of BSUV. In fact, the opposite is observed in the present data (highest BSUV group averages for site 3). This is in accord with our assessment that the possible influence of diuretic application on FDG excretion (and FDG availability during the PET scan) is so small that it is of no concern here.

A further indication that physiological/biological differences between patient groups are not an issue is the fact that no notable intra-site differences of group averaged BSUV were observed over 5 years and three groups at each of the three participating sites, respectively.

A very conservative attitude would be to conclude that observed inter-group differences only provide a strong indication (rather than proof) of underlying differences in scanner calibration, signaling the need for additional quality assurance measures such as further phantom measurements to control or correct the current scanner calibration. In our view, however, it is plausible to accept observed inter-group differences of mean BSUV as directly reflecting differences of the effective scanner calibration as it is operational in real patient data. Dedicated studies, ideally including blood sampling, would be desirable in order to definitely decide whether this assessment is correct.

## Conclusions

Image-based determination of the group-averaged blood SUV in modestly large groups of whole-body FDG-PET investigations is a viable approach for ensuring consistent scanner calibration over time and across different sites. We propose this approach as a quality control and cross-calibration tool augmenting established phantom-based procedures.
